# The effect of Leopold maneuver training with hybrid simulation on nursing students’ stress, Bio-Psychosocial response and stress coping behaviors: a randomized controlled trial

**DOI:** 10.1186/s12912-025-03386-1

**Published:** 2025-07-01

**Authors:** Nevin Utkualp, Eda Unal, Aysel Ozdemir

**Affiliations:** 1 Faculty of Health Sciences, Department of Obstetrics and Gynecology Nursing, Bursa Uludag University, Bursa, 16059 Türkiye; 2 Faculty of Health Sciences, Department of Public Health Nursing, Bursa Uludag University, Bursa, 16059 Türkiye; 3 Faculty of Health Science, Bursa University, Görükle, Bursa, Türkiye

**Keywords:** Leopold maneuvers, Nursing students, Simulation, Stress

## Abstract

**Background:**

Leopold maneuvers are one of the independent roles of nurses to monitor the level of intrauterine development of the fetus. The objective of this study was to evaluate the effects of simulated Leopold maneuver training on safe pregnancy follow-up of nursing students.

**Methods:**

A randomized controlled experimental design study used a questionnaire. The sample consisted of 4th-grade nursing students (*n* = 66). The study was conducted between May-June 2023. Data were collected using a form that included 18 issues The study was conducted in five steps. Participants were randomly assigned to either the experimental or control groups.

**Results:**

The study found that students in the experimental group who received training using hybrid simulation had significantly higher Leopold knowledge (EG = 12.78 ± 3.06; CG = 5.93 ± 1.17), skills (EG = 14.87 ± 2.02; CG = 8.66 ± 2.43), and correct assessment of Leopold maneuvers (EG = 3.09 ± 0.81; CG = 0.51 ± 0.61) compared to students in the control group (*p* < 0.001). Our study found that the Perceived Stress Scale for Nursing Students (EG = 58.96 ± 14.81; CG = 79.15 ± 15.28), Bio-Psycho-Social Response Scale for Nursing Students (EG = 21.81 ± 7.16; CG = 41.36 ± 12.41), and Stress Coping Behaviors Scale for Nursing Students (EG = 46.718 ± 5.57; CG = 37.36 ± 7.04) scores of the group that received simulated Leopold maneuvers training were significantly different from the control group (*p* < 0.001).

**Conclusion:**

Learning through simulation in women’s health nursing education helped students gain Leopold knowledge, skills, and accurate assessments. Simulated education decreased students’ Perceived Stress for Nursing Student, Bio-Psycho-Social Response for Nursing Students’ levels, and increased their Stress Coping Behavior for Nursing Students’ levels. Our study at the ISRCTN registry: ttps://www.isrctn.com/ISRCTN16201085. number and is stated “Retrospectively registered”.date:4 November 2024.

**Supplementary Information:**

The online version contains supplementary material available at 10.1186/s12912-025-03386-1.

## Background

Obstetric nursing requires a variety of basic skills to ensure pregnant health and safety. The skills encompassed in this list are empathy, adaptability, dexterity, manual dexterity, teamwork, and the ability to remain calm in a crisis for the purpose of ensuring safety ( [1, 2]). The fact that students are often pregnant women who experience fear and anxiety in clinical settings requires them to manage the process well. Addressing these concerns can help enhance student performance and the quality of care in the pediatric clinical environment [3]. Clinical practice is an integral part of nursing education. Integration of theoretical knowledge and clinical practice in nursing education is necessary to provide quality care. In the present era, pre-clinical education holds significance owing to the rising enrollment of students in nursing education, the inadequate availability of teaching personnel, and the implementation of measures concerning patient safety. Implementing innovative learning techniques in nursing education offers numerous benefits for student learning. Educational simulations, as a novel pedagogical approach, have the potential to enhance students’ knowledge, enhance their abilities and achievements, and have a positive impact on their interest and motivation [4]. Systematic reviews have demonstrated that simulation training, in particular, can enhance the skills of nursing students, thereby playing a crucial role in enhancing the quality of patient care [5, 6]. Simulation training has been found to support clinical practice and improve clinical and communication skills [7, 8]. Simulation training acts as a bridge between school and clinical practice, allowing the student to improve their knowledge and skills. Simulation training enables students to enhance their learning of hands-on clinical examination and procedural skills by utilizing realistic partial tasks and high-quality simulators prior to engaging with actual patients. According to the literature, it was reported that knowledge scores increased and skills improved in medical and nursing students who received simulation training according to the results of a four-year study in a gynecology clinic [9]. It is also important for students to gain proficiency in theoretical knowledge and skills and gain self-confidence through simulation training [9]. Nursing students face some problems during the nursing interventions they apply to develop and reinforce their skills in the clinical environment [7]. Several challenges arise, such as a scarcity of patients suitable for training, limited availability of clinical situations, and a large student population. Opportunities for students to learn about patients may be limited due to the importance of privacy in women’s health examinations [3]. The applicability of simulation education in nursing education seems to be quite high [10]. With the application of simulation, many nursing students can improve their knowledge, skills, and performance in this field [11]. Thus, when active methods are applied to the teaching-learning process, they offer students a new perspective and increase student motivation and interest [12]. Simulation training provides various educational benefits such as improving nursing knowledge and skills as well as increasing student satisfaction, encouraging critical thinking, and increasing self-confidence [13]. It is crucial for students to be able to link patients and interventions with care goals in order to deliver high-quality nursing care and ensure patient safety. By engaging in this educational opportunity, students can attain a profound level of critical thinking abilities and effortlessly acquire professional skills. It is expected to improve students’ analytical and critical thinking skills by providing them with the opportunity to combine theoretical and practical knowledge [14]. Before going to the clinic, the hybrid simulation technique allows the student to reach professional competencies by developing cognitive, affective, and psychomotor skills in a safe and realistic environment [15]. This training method holds significant importance in the field of clinical nursing education. It allows students to engage in the application of clinical and decision-making skills in scenarios that they may come across. Students learning clinical skills on “real patients” may pose a risk in terms of patient safety and also bring many ethical concerns [16, 17]. Numerous studies have demonstrated that simulation-based learning facilitates the sharing of personal experiences, the identification of clinical knowledge gaps, and the management of clinical issues by nursing students [5, 18, 19]. Besides gaining professional skills, simulation learning is also reported to help students manage pre-clinical stress. The results of a study conducted within the scope of the obstetrics and women’s health nursing course in our country, simulation-based education programs are recommended as they provide good educational practices that allow students to learn by doing and opportunities for repeated practice as well as experience [20]. In other studies, students stated that they found the standardized/simulated patient programs positive and that this training would be useful for them [18, 21]. Since few studies have been conducted on the experiences of nursing students in our country, more information about this teaching method is needed. The objective of this study was to ascertain the impact of Leopold maneuvers training with hybrid simulation on students’ stress, bio-psychosocial response, and stress-coping behavior.

1. Experimental group enhances nursing students` Leopold maneuver knowledge more than the control group.
2. Experimental group enhances nursing students` Leopold maneuver skills more than the control group.
3. Experimental group enhances nursing students` Correct Assessment of the Leopold Maneuver skills more than the control group.
4. The Perceived Stress Scale for Nursing Students’ scores of the students in the experimental group group were lower than those of the control group.
5. The Bio-Psycho-Social Response Scale for Nursing Students’ scores of the students in the experimental group group were lower than those of the control group.
6. The Stress Coping Behaviors Scale for Nursing Students’ scores of the students in the experimental group group were lower than those of the control group.

## Methods

### Study design

In this study, a randomized controlled experimental design was used.

### Sampling

The research was carried out in the Department of Nursing, Faculty of Health Sciences, Bursa Uludağ University. The study was conducted in Bursa Uludag University, Faculty of Health Sciences, Department of Nursing and Health Research and Application Hospital, Department of Obstetrics in May June 2023.

**Fig. 1:**
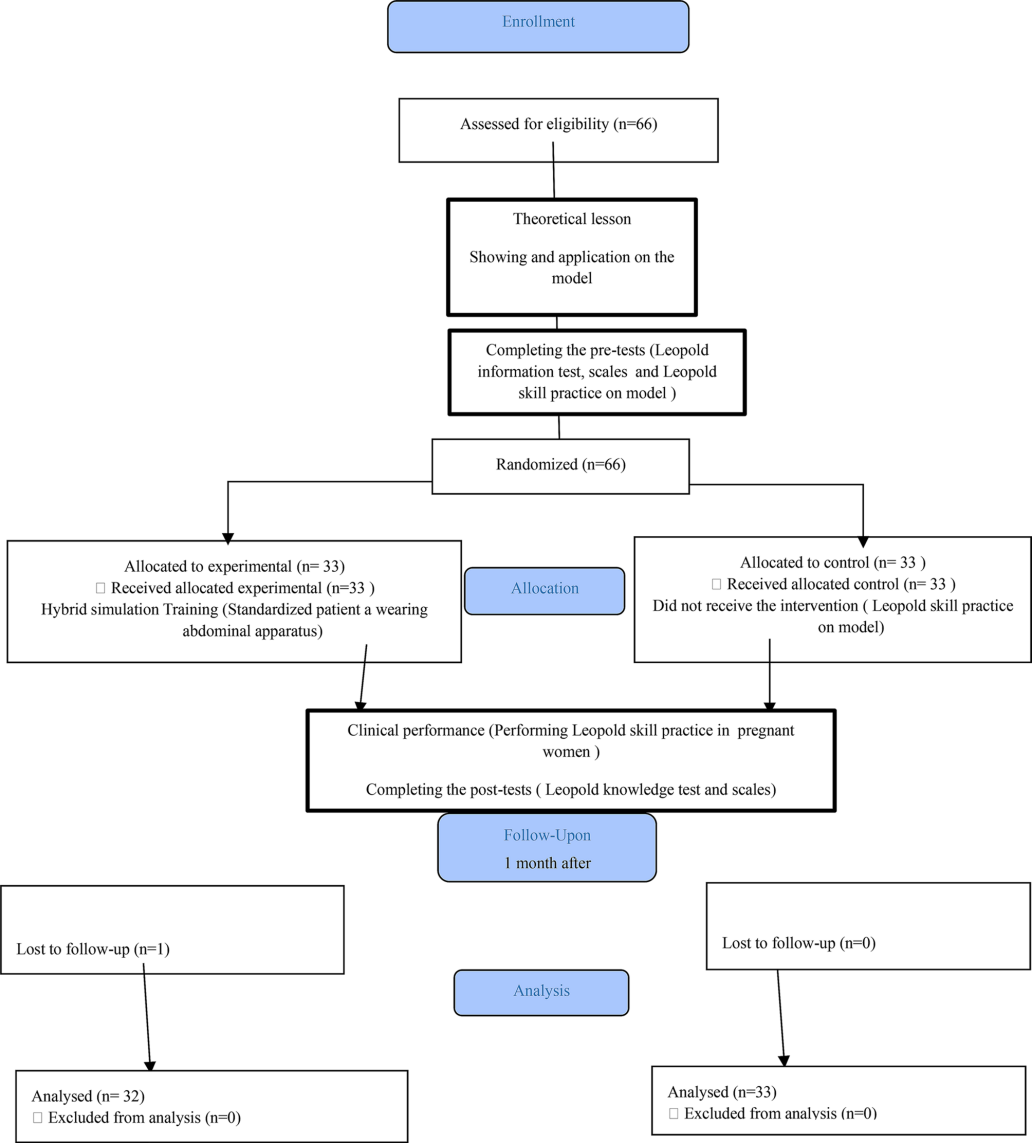
CONSORT flow diagram

The research was carried out with nursing students in the fourth grade (*n* = 66). The inclusion criteria were determined as volunteering and being a 4th-grade nursing student. The exclusion criteria were not volunteering and not being a 4th-grade student. Exposure to practice in the obstetrics department. Before the research was conducted, the sample size was determined to be at least 60 students for 80% power and 0.70 effect size (G*Power Version 3.1.9.7 statistical software). Considering that there would be losses, 10% more students were included in the study (*n* = 66). They were randomized as an experimental group (*n* = 33) and a control group (*n* = 33). The study was conducted with a cohort of 65 students who satisfactorily completed the questionnaire, knowledge test, and scales. One student in the intervention group was excluded from the study due to non-participation in the post-test (Fig. 1).

### Procedures

Voluntary pregnant women without risky pregnancies who come to the obstetrics department.

The study was carried out through a series of five sequential steps:

Step 1: Theoretical information about the Leopold maneuver was given to all students and a demonstration of the Leopold maneuver was performed on a model (50 min). Standard education (theoretical education + demonstration of the Leopold maneuver on a model by the instructor + observation by the instructor while students practice on the model).

Step 2: Descriptive questionnaire form, Leopold information test, and scales (Perceived Stress Scale for Nursing Students, Bio-Psycho-Social Response Scale for Nursing Students, Stress Coping Behaviors Scale for Nursing Students) were sent to the students via WhatsApp and they were asked to fill them out. The ability of the students (*n* = 66) who completed the questionnaire form, knowledge test, and scales completely was evaluated according to the checklist (pre-test). Students do not know which group they are in. One researcher in the study assigned the groups and knew the intervention and control groups. This researcher did not participate in any other part. One researcher did the training. One researcher did the measurements in the clinic. The statistician did not know the experimental and control groups. The two researchers responsible for assessing pre- and posttest knowledge and skills were unaware of the students’ group assignment. A third researcher, who performed the randomization, was aware that the students were assigned to the experimental or control group but was not involved in the training or assessment phases of the study. The students were blinded to whether they were placed in the experimental or control group.

Step 3: The students were randomly assigned to either the experimental or control groups. The process of randomization was used to assign participants to either the experimental group (number 1) or the control group (number 2) based on the numbers they selected from a sealed bag. The students participating in the study consisted of 66 participants in total, including the experimental group (*n* = 33) and the control group (*n* = 33).

Step 4: The experimental group (simulation-based training) underwent training in the Leopold maneuver using hybrid simulation. Students were sent the scenario before the simulation. Students were informed about the standardized patient and environment and the objectives were repeated. For the simulation training, a scenario was designed in which students were asked to evaluate Leopold maneuvers on a pregnant woman[22]. The scenario was given to a standard patient two weeks in advance to allow for adequate preparation. The objectives were clearly explained to the students: communicating with the first mother, providing appropriate positioning, performing the Leopold I Maneuver, determining fundal height and assessing gestational age, performing the Leopold II Maneuver and being able to evaluate the child’s heart sound, performing the Leopold III Maneuver and being able to evaluate the presentation of the fetus, and performing the Leopold IV Maneuver and being able to assess whether the pelvic side of the baby has settled in the pelvis.

The Leopold scenario was performed with a standard patient wearing pregnancy inserts.

Four or five students participated in each session, and the scenario was repeated seven times. Some students acted out the scenario while the remaining students observed their peers’ performances from the analysis room. After the students were prepared, the simulation began with a 5-minute briefing that introduced the scenario summary, learning objectives, expectations, roles, standard patient, and materials. A break was given after all students completed the simulation. Then, with the help of the instructors, the plus/delta method was used during the approximately 30-minute defibrillation session. During the Leopold maneuver training, students provided explanations of the actions performed and the resulting results and identified areas for improvement [19]. The total duration of the simulation-based training for each student was 50 min and consisted of a 5-minute briefing, a 15-minute simulation, and a 30-minute debriefing. The entire simulation process for the intervention group lasted 170 min in total; each of the seven groups completed a 5-minute briefing and a 15-minute simulation, followed by a 30-minute debriefing collectively.

Step 5: A month after the simulation training, the experimental and control groups were evaluated while performing Leopold skill practice in pregnant women in the obstetrics department. Students completed the Leopold knowledge test and scales (Perceived Stress Scale for Nursing Students, Bio-Psycho-Social Response Scale for Nursing Students, and Stress Coping Behaviors Scale for Nursing Students) for the second time (post-test). Step 5 was completed in a total of 8 h.

### Measurement

Leopold Maneuvers Knowledge Scale (0–18): A knowledge test was created by reviewing the literature. The knowledge test comprises 18 multiple-choice questions. One point was awarded for each correct answer, while zero points were awarded for each incorrect answer. The minimum score achievable on the knowledge test is 0, while the maximum score is 18. The knowledge test questions included questions about the process steps of Leopold’s maneuvers, the purpose of performing them, and the evaluation of the data obtained [13, 15].

Leopold Maneuvers Application Skill Control Form (SCF) (0–18**)**: The SCF was prepared by reviewing the literature to measure the application steps. According to the SCF, each correct application was given 1 point and each incorrect/incomplete application was given 0 points. The minimum score achievable as per the SCF is 0, while the maximum score is 18. SCF encompasses the procedural stages of the Leopold maneuver [13, 15, 16].

Situation Assessment Form (SAF) (0–4): The SAF was prepared to determine the student’s ability to correctly evaluate Leopold’s maneuvers. According to the SAF, 1 point was given for the step performed correctly and 0 point was given for the step performed incorrectly/incompletely. The minimum score on the SAF is 0 and the maximum score is 4. The SAF includes the Leopold maneuver knowledge step, the Leopold maneuver application step, the Leopold maneuver data detection step, and the Leopold maneuver results evaluation step [13, 15, 16].

Perceived Stress Scale for Nursing Students (PSSNS): The instrument used to gauge nursing students’ perceived stress levels is the Perceived Stress Scale for Nursing Students. In order to evaluate the validity and reliability of the scale in Turkey, Karaca et al. conducted a study in 2015. Turkey, Karaca et al. conducted a study in 2015 [23]. With six sub-dimensions and a total of 29 items, the 5-point Likert scale goes from 0 (Not stressful for me) to 4 (Very stressful for me). The total score is between 0 and 116. A high score denotes a high degree of stress. Kaiser-Meyer-Olkin was found by Karaca et al. to be 0.94 on the stress scale for nursing students. The calculated chi-square value was statistically significant, according to the perceived stress scale’s Barlett test result [23]. For the felt stress scale, the Cronbach’s alpha reliability coefficient varied from 0.67 to 0.93. Cronbach’s alpha reliability coefficients from the study were 0.937 and 0.940.

Bio-Psycho-Social Response Scale for Nursing Students (BPSSNS): In order to evaluate the validity and reliability of the scale in Turkey, Karaca et al. conducted a study in 2015. Turkey, Karaca et al. conducted a study in 2015 [23]. There are 21 questions total on the 5-point Likert scale (4 = always experienced − 0 = never experienced), which is divided into 3 sub-dimensions. The range of the total score is 0 to 84. The biopsychosocial responses to stress that nursing students experience during clinical practice are included in this scale. An elevated score indicates a noteworthy degree of biopsychosocial responses to stress [23]. The reliability coefficient of Cronbach’s alpha was determined to be 0.932 and 0.905 in the study.

Stress Coping Behaviors Scale for Nursing Students (SCBSNS**)**: In 2015, Karaca et al. conducted an assessment of the scale’s validity and reliability in Turkey. Turkey, Karaca et al. conducted a study in 2015 [23]. With four sub-dimensions and nineteen items, the 5-point Likert scale (4 = always experienced − 0 = never experienced) is composed. The overall score is between 0 and 76. This measure encompasses coping mechanisms applied in high-stress educational and professional contexts. A high score denotes the application of coping mechanisms to a high degree under stressful circumstances [23]. The reliability coefficients for Cronbach’s alpha in this study were 0.672 and 0.714.

### Statistical analysis

The statistical software program SPSS 23.0 was used to examine the data. A t-test was utilized to compare two groups whose data followed a normal distribution, while the Whitney U test was applied to data whose distribution was non-normal. The change value (Difference score = Post-test measurement - pretest measurement) was computed and compared between the groups in a repeated measures analysis. Dependent samples were compared using the dependent sample t-test and the Wilcoxon Sign test. To compare categorical data, the Fisher’s Exact Test and Chi-square test were used. A significant threshold of α = 0.05 was set.

## Results

Students in the control group were 21.81 ± 0.88 years old, while students in the experimental group were 21.59 ± 0.71 years old on average. 84% of participants in the experimental group were female, compared to 85% in the control group. Table 1 shows that there was no statistically significant difference in age or gender across the groups (*p* > 0.05).

**Table 1:** Comparison of students’ descriptive characteristics

	Experimental Group (n=32)		Control Group (n= 33)		Test Statistics	*p*-value
Age	21. 59 ± 0.71		21.81 ± 0.88		458.500*	0.326
Gender	**n**	**%**	**n**	**%**		
Female	27	84	28	85		
Male	5	16	5	16	1.000**	0.628

Descriptive statistics are given as mean ± standard deviation and frequency (%)

*Mann Whitney U Testi, **Fisher’s Ecact Test

The average pre-test Leopold knowledge scores did not differ statistically significantly between the groups (*p* = 0.408). The mean post-test Leopold knowledge score of the students in the experimental group was 12.78 ± 3.06, which was significantly higher than the mean post-test Leopold knowledge score of the students in the control group, which was 5.93 ± 1.17 (*p* < 0.001) (Table 2).

**Table 2:** Comparison of Leopold knowledge, skill, and accurate evaluation scores between and within groups

	Experimental Group (32)	Control Group (33)	Test Statistics	*p*-value
**Leopold Knowledge (0–18 )**				
Pre-test	5.37 ± 0.94	5.63 ± 0.85	468.500*	0.408
Post-test	12.78 ± 3.06	5.93 ± 1.17	21.000*	**< 0.001**
(Δ)	7.40 ± 3.15	0.30 ± 1.13	26.00*	**< 0.001**
Test Statistics	-4.866***	-1.467***		
*p-*value	**< 0.001**	0.142		
**Leopold Skill (0–18 )**				
Pre-test (model)	9.34 ± 3.09	8.66 ± 2.39	0.988**	0.327
Post-test (Clinical setting)	14.87 ± 2.02	8.66 ± 2.43	11.158**	**< 0.001**
(Δ)	5.53 ± 2.78	0.00 ± 0.35	2.000**	**< 0.001**
Test Statistics	-4.945****	0.00****		
*p-*value	**< 0.001**	1.00		
**Correct Assessment of the Leopold Maneuver (0–4)**				
Model	0.84 ± 0.76	0.78 ± 0.78	505.500*	0.752
Clinical setting	3.09 ± 0.81	0.51 ± 0.61	14.500*	**< 0.001**
(Δ)	2.25 ± 1.10	-0.27 ± 0.67	40.500*	**< 0.001**
Test Statistics	-4.808***	-2.183***		
*p-*value	**< 0.001**	**0.029**		

Δ = Post test-Pre test

Descriptive statistics are given as mean ± standard deviation

* Mann Whitney U Test

**Independent Sample T-Test

***Wilcoxon Test

**** Dependent Sample T-Test

The mean pre-test Leopold skill ratings for each group did not differ statistically significantly (*p* = 0.327). The mean post-test Leopold knowledge score of the students in the experimental group was 12.78 ± 3.06, which was significantly higher than the mean post-test Leopold knowledge score of the students in the control group, which was 5.93 ± 1.17 (*p* < 0.001) (Table 2).

The groups’ mean scores for correctly evaluating the Leopold movements from the pre-test did not differ statistically significantly (*p* = 0.752). The mean post-test for correctly evaluating Leopold moves score of the students in the experimental group was 3.09 ± 0.81, which was significantly higher than the mean post-test for correctly evaluating Leopold moves score of the students in the control group, which was 0.51 ± 0.61 (*p* < 0.001) (Table 2).

The average PSSNS scores of the groups before to the test did not differ significantly (*p* = 0.545). The post-test PSSNS mean scores showed a statistically significant difference between the groups (*p* < 0.001). The mean post-test PSSNS mean score of the students in the experimental group was 58.96 ± 14.81, which was significantly higher than the mean post-test PSSNS mean score of the students in the control group, which was 79.15 ± 15.28 (*p* < 0.001) (Table 3).

**Table 3:** Comparison of perceived stress, bio-psycho-social response and stress coping behaviors scale for nursing students scores between and within groups

	Experimental Group (*n* = 32)	Control Group (*n* = 33)	Test Statistics	*p*-value
**Perceived Stress Scale for Nursing Students (PSSNS) (0-116)**				
Pre-Test	76.96 ± 19.66	79.63 ± 15.51	-0.608*	0.545
Post-test	58.96 ± 14.81	79.15 ± 15.28	-5.403*	**< 0.001**
(Δ)	-18.00 ± 13.88	-0.48 ± 20.65	4.000*	**< 0.001**
Test statistic	-7.334**	-0.135**		
*p-*value	< 0.001	0.894		
**Bio-Psycho-Social Response Scale for Nursing Students (BPSSNS) (0–84)**				
Pre-Test	29.04 ± 13.68	35.90 ± 18.01	-1.635*	0.107
Post-test	21.81 ± 7.16	41.36 ± 12.41	-7.745*	**< 0.001**
(Δ)	-7.59 ± 8.66	5.45 ± 11.68	-5.101*	**< 0.001**
Test statistic	-4.955**	-2.564**		
*p-*value	< 0.001	0.010		
**Stress Coping Behaviors Scale for Nursing Students (SCBSNS) (0–79)**				
Pre-Test	37.75 ± 8.11	39.72 ± 8.76	0.943*	0.349
Post-test	46.718 ± 5.57	37.36 ± 7.04	7.04*	**< 0.001**
(Δ)	8.96 ± 9.21	-2.36 ± 10.965	5.924*	**< 0.001**
Test Statistics	5.503**	-1.240**		
*p-*value	< 0.001	0.224		

Δ = Post test-Pre test

Descriptive statistics are given as mean ± standard deviation

* Independent Sample T-Test

**Dependent Sample T-Test

The mean pretest BPSSNS scores did not differ statistically significantly across the groups (*p* = 0.107). The post-test BPSSNS mean scores showed a statistically significant difference between the groups (*p* < 0.001). The mean post-test BPSSNS mean score of the students in the experimental group was 21.81 ± 7.16, which was significantly higher than the mean post-test BPSSNS mean score of the students in the control group, which was 41.36 ± 12.41 (*p* < 0.001) (Table 3).

SCBSNS pretest averages did not change statistically significantly between the groups (*p* = 0.349). The groups’ mean scores on the post-test SCBSNS showed a statistically significant difference(*p* < 0.001). The mean post-test SCBSNS mean score of the students in the experimental group was 46.718 ± 5.57, which was significantly higher than the mean post-test SCBSNS mean score of the students in the control group, which was 37.36 ± 7.04 (*p* < 0.001) (Table 3).

## Discussion

### Clinical skills and theoretical knowledge enhancement

Our study demonstrated that students in the experimental group who received hybrid simulation-based training achieved significantly higher mean scores in clinical skills compared to those in the control group. Furthermore, simulation-based learning contributed to measurable improvements in theoretical knowledge and self-confidence. Students also showed meaningful progress between pre- and post-training assessments, reflecting the effectiveness of the educational approach. Participants reported that the hybrid simulation model significantly enhanced their clinical competencies. No statistically significant differences were found between groups in terms of gender or age (Table 1). Although our study did not reveal gender-based differences, previous research has indicated that female students often report higher levels of empathy and psychological resilience in clinical care [2]. The increased satisfaction of female students with simulation-based learning, as noted in earlier studies, aligns with the generally positive perceptions of simulation reported in our findings [8, 16, 24]. A study conducted in Ethiopia found that midwifery students who received simulation-based training for skill acquisition demonstrated significantly greater clinical competence than those who were trained using traditional instructional methods. The opportunity to repeatedly perform procedures in a low-risk environment contributed to both skill development and higher levels of student satisfaction [25]. Similarly, previous research has demonstrated that experiential learning involving birth and pregnancy scenarios plays a crucial role in developing professional competencies among nursing students [10].In our study, students in the hybrid simulation group also achieved higher mean scores in both Leopold maneuver knowledge and performance accuracy, suggesting that simulation-based education enhances procedural understanding and precision in obstetric assessments. This outcome is supported by studies reporting that simulation-based experiential learning fosters critical thinking and increases learner satisfaction [14]. For instance, Holmström et al. (2011) found that students who participated in simulation training reported greater confidence in their ability to perform vaginal deliveries compared to those who received only traditional education. Moreover, these students achieved significantly higher scores on both written and oral examinations following practical sessions [26]. Simulation-based training has also been recognized as an effective method for nurturing a broad range of essential nursing competencies, including clinical empathy, adaptability, psychomotor skills, teamwork, leadership, time management, and crisis response [14]. Among midwifery students, those who received simulation training reported higher satisfaction and self-confidence than those who were educated using conventional antenatal lecture-based approaches [27].

Underlying Mechanisms of Hybrid Simulation.

The effectiveness of hybrid simulation in reducing students’ stress and enhancing clinical competence may be explained through several interconnected mechanisms. First, simulation environments provide a psychologically safe space where students can engage in clinical tasks without fear of harming real patients, thus reducing performance anxiety and allowing for emotional regulation particularly in high-stakes procedures such as obstetric assessments [28, 29]. This safety fosters emotional regulation and promotes confidence, particularly in high-stakes procedures such as the Leopold maneuvers. Moreover, the repetitive and hands-on nature of simulation supports the principles of deliberate practice [30], allowing learners to refine psychomotor and decision-making skills with immediate feedback. The hybrid model, by integrating theoretical instruction with experiential learning, also enhances cognitive retention and skill transfer to real clinical settings. These mechanisms are supported by experiential learning theory [31], which emphasizes the importance of active engagement and reflective practice in achieving meaningful learning outcomes.Consistent with these results, another study reported that students performed Leopold maneuvers with greater ease and confidence, which in turn contributed to decreased anxiety and emotional tension.

Stress Reduction and Coping Skills.

Our findings revealed that students in the hybrid simulation group experienced reduced stress levels and demonstrated stronger coping skills compared to the control group. These outcomes are supported by other studies indicating that simulation-based training contributes to increased confidence in performing complex tasks such as the Leopold maneuvers, which in turn alleviates anxiety and emotional tension [27, 32].A likely explanation for this effect is that simulation provides a structured, low-risk environment where students can practice clinical procedures repeatedly, free from the immediate pressures and responsibilities of real patient care. These psychologically safe settings allow learners to manage stress more effectively while enhancing clinical accuracy through guided repetition and feedback.In contrast, Ahmadi et al. reported that midwifery students experienced considerable fear and anxiety prior to clinical placements, particularly due to concerns about harming pregnant women. This fear was found to reduce motivation, increase stress, and hinder the formation of professional identity [7, 17].Numerous studies have emphasized that simulation and case-based instruction prior to clinical practice play a crucial role in preparing students both cognitively and emotionally. Such approaches not only enhance professional competencies but also reduce pre-clinical stress and improve coping abilities in real-life healthcare environments [14, 17]. Similarly, a national study reported that while simulation training reduced students’ stress and clinical dissatisfaction, it had no significant effect on self-confidence levels [20].

These findings are further supported by a study conducted in a gynecology clinic, where both medical and nursing students showed strong interest in simulation-based learning. The shared experience positively influenced their professional behaviors through interprofessional collaboration and mutual engagement [9]. Many studies have demonstrated that simulation-based education enhances educational outcomes across diverse healthcare disciplines. It has been associated with improvements in clinical reasoning, communication, interprofessional collaboration, and learner satisfaction [17, 20]. National research also confirms that standardized and simulated patient programs are positively received by students, who report that these methods increase their preparedness for real clinical environments and reduce anxiety during patient interaction [15, 20, 21]. Overall, simulation-based education is widely recognized as an effective and interactive instructional strategy that supports the development of clinical competencies and improves student engagement. Its integration into nursing curricula addresses both cognitive and affective domains of learning and is considered an essential component of contemporary nursing education [33][34].

### Limitations and future research directions

This study has several limitations that should be acknowledged. First, the research was conducted at a single nursing school in Türkiye, which may limit the generalizability of the findings to other institutions and geographic regions. Second, the follow-up period was relatively short, and participation was affected by scheduling conflicts between the study sessions and students’ academic commitments. Third, the findings reflect students’ perceptions of the simulation experience at a single point in time, which may not capture long term impacts on academic performance or professional adjustment.

Additionally, it is possible that the high level of engagement and awareness among senior students contributed to the elevated outcomes observed, potentially influencing the general applicability of the results. Future research is recommended to include multi-center and longitudinal designs to assess the sustained impact of hybrid simulation on clinical competence, stress management, and professional identity development across diverse nursing student populations.

## Conclusions

In our study has found that using simulation-based education in women’s health nursing has a positive impact on students’ knowledge, and stress management. This suggests that incorporating simulation-based education into nursing student learning can be beneficial.

The findings of this study show that simulation-based education in women’s health nursing has a positive effect on students’ knowledge acquisition, and stress management. Therefore, we think that the use of simulation-based education as a pedagogical approach that facilitates learning in the education of nursing students will be beneficial for those who participate in such studies.

## Electronic supplementary material

Below is the link to the electronic supplementary material.

Supplementary Material 1

Supplementary Material 2

## Data Availability

No datasets were generated or analysed during the current study.

## Abbreviations

SCF: Skill Control Form
SAF: Situation Assessment Form
PSSNS: Perceived Stress Scale for Nursing Students
BPSSNS: Bio-Psycho-Social Response Scale for Nursing Students
SCBSNS: Stress Coping Behaviors Scale for Nursing Students

## References

[1] Evli M. Compassion fatigue, empathy, and emotional contagion in nursing students. J Educ Res Nurs. 2023;20:105–10.

[2] Kumara A, Ameh C. Starthere-principles of effective undergraduate training. Best Pract Res Clin Obstet Gynaecol. 2022;Apr:80:114–25. 10.1016/j.bpobgyn.2021.11.010 34952793

[3] Zengin H, Fidanci BE. Effect of working with a standardized pediatric patient on the skills of nursing students in Preparing children for a medical procedure. Clin Simul Nurs. 2024;87:101488.

[4] Khoshbakht-PishkhaniM, Javadi-Pashaki N, Sayad Noveiry MJ, F Asgar.Simulated Nursing Grand Rounds in The Clinical Education of Postgraduate Nursing Students. Res Square Platf. 2023;13:1–9. 10.21203/rs.3.rs-3020243/v1.

[5] Cant RP, Cooper SJ. Use of Simulation-Based learning in undergraduate nurse education: an umbrella systematic review. Nurse Educ Today. 2017;49:63–71. 10.1016/j.nedt.2016.11.015. 27902949 10.1016/j.nedt.2016.11.015

[6] Haddeland K, Slettebø A, Carstens P, Fossum M. Nursing students managing deteriorating patients: a systematic review and meta-analysis. Clin Simul Nurs. 2018;21:1–15. 10.1016/j.ecns.2018.05.001.

[7] Ahmady S, Khajeali N, Afshari P. Explaining midwifery students’ experience of their first Atten-dance in clinical skill centers: A content analysis study. J Med Educ. 2023;22:137135.

[8] Olaussen C, Aase I, Petter L, Christine J, Simen A, Steindal. Supplementing clinical practice in nursing homes with simulation training: A qualitative study of nursing students’. Experiences SAGE Open Nurs. 2020;6:1–11. 10.1177/2377960820981786 PMC8832293 35155765

[9] Ogunyemi D, Christopher H, Vallie S, Thomas M. Evolution of an obstetrics and gynecology interprofessional Simulation-based education session for medical and nursing students. Medicine. 2020;99:43. 10.1097/MD.0000000000022562 PMC7581067 33120744

[10] Danna VA, Bedwell C, Chimwaza A, Chisuse I, Lyangenda K, Petross C, et al. Promoting respectful maternal and newborn care using the dignity game: A quasi-experi-mental study. Nurse Educ Pract. 2023;66:103519. 36442392 10.1016/j.nepr.2022.103519 PMC9912051

[11] Uslu Y, Ünver V, Kocatepe V, Karabacak Ü. Example of a simulation design in nursing education: safe chemotherapy administration. FNJN. 2019;27:2147–4923. 10.5152/FNJN.2019.18081 PMC8127577 34267983

[12] Coelho J, Moreno A, Roldán MJ, Sequeira C, Sampaio F. Perspectives of Adult Patients with Mental Health Disorders on the Relationship with Nurses: A Focus Group Study. BMC Nurs.2024;23:9. 10.1186/s12912-023-01663-5.PMID: 38163914. 10.1186/s12912-023-01663-5 PMC10759621 38163914

[13] Eun JK, Kyung MC, Sung SS. Child nursing simulation scenario content analysis: a directed qualitative content analysis. Clin Simul Nurs. 2024;87:101488.

[14] Chang CY, Kao CH, Hwang GJ, Lin FH. From experiencing to critical thinking: A contextual Game-Basedlearning approach to improving nursing students’performance in Electrocar-Diogram training. Education Tech Research Dev. 2020;68:1225–45.

[15] Atan ÜŞ, Şatır DG, Öztürk R, Kavlak O,Saruhan A, Güneri ES. The eff.ect of using high fidelity birthing simulator on satisfaction and performance of nursing students in developing obstetric skills.fnjn 2019; 27:1–16. 10.26650/FNJN341399 PMC8127588 34267958

[16] Agha S, Alhamrani AY, Khan MA. Satisfaction of medical students with simulation based learning. Saudi Med J. 2015;36:731–6. 10.15537/smj.2015.6.11501. 25987117 10.15537/smj.2015.6.11501 PMC4454909

[17] Kaya A, Düzgün MV, Boz İ. The relationship between professional values, ethical sensitivities and caring behaviors among nursing students: A structural equation modeling approach. Nurse Educ Pract. 2023;70:103–676. 10.1016/j.nepr.2023.103676. 10.1016/j.nepr.2023.103676 37276776

[18] Karaçay P. Göktepe N.The use of simulation method in the education of nursing students before their first clinical practice.Paper presented at the Congress on Clinical and Communication Skills Training in Health Sciences.2011.

[19] Özdemir A, Ünal E. The effect of breast Self-Examination training on nursing students by using Hybrid-Based simulation on knowledge, skills, and ability to correctly evaluate pathological findings: randomized controlled study. Nurse Educ Pract. 2023;66:103530. 36462274 10.1016/j.nepr.2022.103530

[20] Erenel AS, Yaman SŞ,Uzun AM, Gürcüoğlu AS, Ünal TF, Uçakcı AC. Effect of Scenario- based simulation training on the obstetrics and gynecology nursing clinical practicum. J Nurs Res. 2021;29:42. 10.1097/jnr.0000000000000417. 10.1097/jnr.0000000000000417 33395173

[21] Terzioğlu F, Yücel Ç, Koç G, Şimşek Ş,Yaşar BN, Şahan FU, et al. A new strategy in nursing education: from hybrid simulation to clinical practice. Nurse Educ Today. 2016;39:104–8. 10.1016/j.nedt.2016.01.009. 27006040 10.1016/j.nedt.2016.01.009

[22] INACSL Standards Committee. INACSL standards of best practice: simulation design. Clin Simul Nurs. 2016;12:5–12.

[23] Karaca A, Yıldırım N, Ankaralı H, Açıkgöz F, Akkuş D. Hemşirelik öğrencileri İçin Algılanan stres, Biyo-psiko-sosyal Cevap ve stresle Başetme Davranışları Ölçeklerinin türkçe’ye Uyarlanması. J Psychiatric Nurs. 2015;6(1):15–25.

[24] Cheng A, Lockey A, Bhanji F, Lin Y, Hunt EA, Lang E. The use of High-Fidelity manikins for advanced life support Training—A systematic review and meta-Analysis. Resusc 2015, 93, 142–9. 10.1016/j.resuscitation.2015.04.004 10.1016/j.resuscitation.2015.04.004 25888241

[25] Jamie AH, Mohammed AS. With Simulation-Based education among bachelor of midwifery students in public universities andcolleges in Harar and dire Dawa cities, Ethiopia. Eur J Midwifery. 2019;31:3–19. 10.18332/ejm/113132. 10.18332/ejm/113132 PMC7839129 33537598

[26] Holmstrom SW, Downes K, Mayer JC, Learman LA. Simulation training in an obstetric clerkship: A randomized controlled trial. Obstet Gynecol. 2011;118:649–54. 21860296 10.1097/AOG.0b013e31822ad988

[27] Kalyoncu E. The effect of teaching methods used in leo:pold manneuts education on satisfaction of midwifery students. Master Thesis 2019.Istanbul Medipol University. Graduate School of Health Sciences. Istanbul.

[28] Basak T, Demirtas A, Iyigun E. The effect of simulation based education on patient teaching skills of nursing students: A randomized controlled study. J Prof Nurs. 2019;35(5):417–24. 10.1016/j.profnurs.2019.02.004. Epub 2019 Feb 8. PMID: 31519347. 31519347 10.1016/j.profnurs.2019.02.004

[29] Oliveira Silva G, Oliveira FSe, Coelho ASG, Fonseca LMM, Vieira FVM, Campbell SH, Aredes N. D. A. Influence of simulation design on stress, anxiety and self-confidence of nursing students: systematic review with meta-analysis. J Clin Nurs. 2023;32:5668–92. 10.1111/jocn.16681. 36894868 10.1111/jocn.16681

[30] Ericsson KA. Deliberate practice and acquisition of expert performance: A general overview. Acad Emerg Med. 2008;15(11):988–94. 10.1111/j.1553-2712.2008.00227.x. 18778378 10.1111/j.1553-2712.2008.00227.x

[31] Kolb DA. Experiential learning: experience as the source of learning and development. Englewood Cliffs, NJ: Prentice Hall; 1984. http://academic.regis.edu/ed205/Kolb.pdf.

[32] Coelho A, Parola V, Cardoso D, Duarte S, Almeida M. The use of the aged simulation suit in nursing students: a scoping review. J Nurs Referência Revista De Enfermagem Referência. 2017;4:147–58.

[33] Alamrani MH, Alammar KA, Alqahtani SS, Salem OA. Comparing the effects of Simulation-Based and traditional teaching methods on the critical thinking abilities and Self-Confidence of nursing students. J Nurs Res. 2018;26:152–7. 10.1097/jnr.0000000000000231. 29016466 10.1097/jnr.0000000000000231

[34] Doolen J, Mariani B, Atz T, Horsley TL, O’Rourke J, McAfee K. High-fidelity Simulation in Doolen, J.; Mariani, B.; Atz, T.; Horsley, TL.; O’Rourke, J.; McAfee, K. High-fidelity Simulation in Undergraduate Nursing Education: A Review of Simulation Reviews. Clinical Simulation in Nursing 2016, 12, 290–302.

