# Supplementary material for: The effect of Leopold maneuver training with hybrid simulation on nursing students’ stress, Bio-Psychosocial response and stress coping behaviors: a randomized controlled trial
Source: BMC Nurs. 2025 Jul 1;24:731. doi: 10.1186/s12912-025-03386-1 (PMC12211145; doi:10.1186/s12912-025-03386-1)
Supplement: Supplementary file 2 — Supplementary Material 2 [file 12912_2025_3386_MOESM2_ESM.pdf]

SURVEY

1.Age:

2.Gender: 1. Female 2. Male

Leopold Maneuvers Knowledge

1.I. What is the purpose of the Leopold maneuver?

a. Determination of fundus height

b. Determination of which part of the baby is presenting

c. Determination of whether the baby’s head is above or below the pubic symphysis

d. Determination of which side of the mother the baby's back is on

e. Determination of the degree to which the presenting part has settled into the pelvis

2. II. What is the purpose of the Leopold maneuver?

a. Determination of fundus height

b. Determination of which part of the baby is presenting

c. Determination of whether the baby’s head is above or below the pubic symphysis

d. Determination of which side of the mother the baby's back is on

e. Determination of the degree to which the presenting part has settled into the pelvis

3. III. What is the purpose of the Leopold maneuver?

a. Determination of fundus height

b. Determination of which part of the baby is presenting

c. Determination of whether the baby’s head is above or below the pubic symphysis

d. Determination of which side of the mother the baby's back is on

e. Determination of the degree to which the presenting part has settled into the pelvis

4. IV. What is the purpose of the Leopold maneuver?

a. Determination of fundus height

b. Determination of which part of the baby is presenting

c. Determination of whether the baby’s head is above or below the pubic symphysis

d. Determination of which side of the mother the baby's back is on

e. Determination of the degree to which the presenting part has settled into the pelvis

5. What is the purpose of Leopold maneuver 5?

a. Determination of fundus height

b. Determination of which part of the baby is presenting

c. Determination of whether the baby's head is above or below the pubic symphysis

d. Determination of which side of the mother the baby's back is on

e. Determination of the degree to which the presenting part has settled into the pelvis

6. What position do you place the pregnant woman in to perform Leopold's maneuvers?

a. Sims position b. Lithotomy position c. Dorsal recumbent position d. Supine position

e. Lateral position

7. From which month of pregnancy can Leopold's maneuvers be performed?

a. 2nd month b. 3rd month c. 4th month d. 5th month e. 6th month

8. When can the baby's heart sounds be heard?

a.2nd month b.3rd month c.4th month d.5th month e.6th month

9. What is the fetal heart rate in the womb?

a. 90-120 bpm b. 120-160 bpm c. 80-130 bpm d. 100-140 bpm e. 90-150 bpm

10.At 36 weeks of pregnancy, where is the fundal height located?

a. 3 finger-widths above the pubic symphysis

b. At the level of the belly button

c. At the level of the ribs

d. Between the belly button and the xiphoid process

e. 3 finger-widths below the belly button

11. At 20 weeks of pregnancy, where is the fundal height located?

a. 3 finger-widths above the pubic symphysis

b. At the level of the belly button

c. At the level of the ribs

d. Between the belly button and the xiphoid process

e. 3 finger-widths below the belly button

12. At 32 weeks of pregnancy, where is the fundal height located?

a. 3 finger-widths above the pubic symphysis

b. At the level of the belly button

c. At the level of the ribs

d. Between the belly button and the xiphoid process

e. 3 finger-widths below the belly button

13. At 24 weeks of pregnancy, where is the fundal height located?

a. 3 finger-widths above the pubic symphysis

b. At the level of the belly button

c. At the level of the ribs

d. Between the belly button and the xiphoid process

e. 3 finger-widths below the belly button

14. At 16 weeks of pregnancy, where is the fundal height located?

a. 3 finger-widths above the pubic symphysis

b. At the level of the belly button

c. At the level of the ribs

d. Between the belly button and the xiphoid process

e. 3 finger-widths below the belly button

15. Which Leopold maneuver is performed by placing one hand's thumb and fingers as far apart as possible over the pubic symphysis?

a. I. Leopold maneuver

b. II. Leopold maneuver

c. III. Leopold maneuver

d. IV. Leopold maneuver

e. V. Leopold maneuver

16. Which Leopold maneuver is performed by placing your fingers on the upper part of the abdomen, spreading them to the sides with the fingertips close together?

a. I. Leopold maneuver

b. II. Leopold maneuver

c. III. Leopold maneuver

d. IV. Leopold maneuver

e. V. Leopold maneuver

17. Which Leopold maneuver is performed by gently placing both hands on the lower part of the abdomen and into the pelvis?

a. I. Leopold maneuver

b. II. Leopold maneuver

c. III. Leopold maneuver

d. IV. Leopold maneuver

e. V. Leopold maneuver

18. Which Leopold maneuver is performed by placing both hands on either side of the abdomen, keeping one hand steady while palpating with the other hand?

a. I. Leopold maneuver

b. II. Leopold maneuver

c. III. Leopold maneuver

d. IV. Leopold maneuver

e. V. Leopold maneuver

Leopold Maneuver Steps Control List<br>Skill Control Form	Did	Did not
1. Wash your hands and, if possible, warm them.		
2. Explain the procedure to the pregnant woman.		
3. Place the woman in a dorsal recumbent position and expose the abdomen.		
4. Be face to face with the pregnant woman.		
5. Place your fingers on the upper part of the abdomen, spreading them to the sides with fingertips close together.		
6. Palpate the fundus with your fingertips. Determine its height.		
7. Turn your face toward the mother.		
8. Place both hands on either side of the abdomen, keeping one hand steady while palpating with the other hand.		
9. Locate the position of the back.		
10. Turn your face toward the mother.		
11. With one hand, place your thumb and fingers as far apart as possible over the pubic symphysis.		
12. Determine what part is presenting.		
13. Turn your back to the pregnant woman.		
14. Gently place both hands on the lower abdomen and into the pelvis.		
15. Determine the condition of the presenting part.		
16. Maintain communication with the pregnant woman throughout the procedure.		
17. Inform the woman about the examination findings.		
18. Record the findings.		

Situation Assessment Form (SAF)

Correct Assessment of Leopold Maneuvers

**Leopold 1**	**True**	**False**
At 16 weeks of pregnancy, the fundus is felt 3 finger-widths above the pubic symphysis.		
At 20 weeks of pregnancy, the fundus is felt 3 finger-widths below the belly button.		
At 24 weeks of pregnancy, the fundus is felt at the level of the belly button.		
At 28 weeks of pregnancy, the fundus is felt 3 finger-widths above the belly button.		
At 32 weeks of pregnancy, the fundus is felt between the belly button and the xiphoid process.		
At 36 weeks of pregnancy, the fundus is in contact with the sternum.		
At 40 weeks of pregnancy, the fundus has dropped 1-2 finger-widths below the sternum.		
**Leopold 2**		
Fetal heart sounds		
**Leopold 3**		
Fetal presentation		
**Leopold 4**		
Proper engagement of the baby in the pelvis		
